# A metric space approach to the information channel capacity of spike trains

**DOI:** 10.1186/1471-2202-12-S1-P152

**Published:** 2011-07-18

**Authors:** James B Gillespie, Conor J Houghton

**Affiliations:** 1 School of Mathematics, Trinity College Dublin, Dublin, Ireland

## 

A novel method is presented for calculating the information channel capacity of spike trains. This method works by fitting a χ-distribution to the distribution of distances between responses to the same stimulus: the χ-distribution is the length distribution for a vector of Gaussian variables. The dimension of this vector defines an effective dimension for the noise and by rephrasing the problem in terms of distance based quantities, this allows the channel capacity to be calculated. As an example, the capacity is calculated for a data set recorded from auditory neurons in zebra finch.

